# Mental health and stigma in persons affected by Hansen’s disease and their families in rural Sitapur, India

**DOI:** 10.1371/journal.pmen.0000475

**Published:** 2025-11-06

**Authors:** Tosca Van Hoorde, Matthew Willis, Srilekha Penna, Ewelina Julia Barnowska, Sophie C. W. Unterkircher, Fabian Schlumberger, Anil Fastenau

**Affiliations:** 1 Department of Health, Ethics and Society, Faculty of Health, Medicine and Life Sciences, Maastricht University, Maastricht, the Netherlands; 2 German Leprosy and Tuberculosis Relief Association India (GLRA India), New Delhi, India; 3 Department of Global Health, Institute of Public Health and Nursing Research, University of Bremen, Bremen, Germany; 4 School of Medicine, Dentistry & Biomedical Sciences, Queen’s University Belfast, Belfast, United Kingdom; 5 Marie Adelaide Leprosy Center (MALC), Karachi, Pakistan; 6 German Leprosy and Tuberculosis Relief Association (GLRA/DAHW), Wurzburg, Germany; 7 Heidelberg Institute of Global Health (HIGH), Medical School, Heidelberg University, Heidelberg, Germany; PLOS: Public Library of Science, UNITED KINGDOM OF GREAT BRITAIN AND NORTHERN IRELAND

## Abstract

Psychological problems are common among persons affected by Hansen’s disease, a chronic but treatable disease that remains endemic in India despite available and effective treatment. Stigmatisation can also lead to delayed care-seeking, which can impede eradication efforts and negatively impact the physical and psycho-social wellbeing of the affected person and their family. This study aimed to examine how stigma in society affect the mental health of persons with Hansen’s disease and their families in endemic villages of Sitapur, India. Semi-structured interviews were conducted to explore how different types of stigma experiences (internalised, anticipated, experienced and perceived) and stigma practices (stereotypes, prejudice, discriminatory behaviour) form in society and affect the mental health of persons affected by Hansen’s disease and their family members. The study was carried out in collaboration with GLRA India and conducted in an endemic district of India, Sitapur. In total, 22 participants were included with a 1:1 of persons affected and family members. Data was analysed via thematic pattern analysis using the Health Stigma and Discrimination (HSD) framework as a structure. Stereotypes and other negative beliefs about Hansen’s disease persisted, and persons affected by the disease and the interviewed family members experienced all types of stigma. Symptoms of anxiety and depression were commonly reported among both patients and family members. Misconceptions about the disease appeared prevalent among patients, families, doctors, and the community. Marriage opportunities were impacted, though segregation was limited. Non-disclosure and self-distancing emerged as coping strategies. The study confirmed the presence of stigma and mental health issues among those affected and their families. Despite misconceptions and fear of spread, family support was high which may serve as a counterbalance to the negative effects of stigma. Education and awareness are essential to combat stigma and improve health outcomes.

## Author summary

Hansen’s disease, commonly known as leprosy, is a chronic but treatable condition that remains endemic in parts of India, including Sitapur. Despite the availability of effective treatment, public health challenges persist, particularly due to stigma. Stigmatization of Hansen’s disease can instil a fear of discrimination in affected persons and can lead to delays in seeking care, hampering eradication efforts. There is evidence to show that depression and anxiety are vastly more prevalent in persons affected by Hansen’s disease compared to non-affected persons. However, the effects of stigma on the mental and social well-being of persons affected by the disease, and their families, remains unclear. This study aimed to explore how stigma influences the mental health of persons affected by Hansen’s disease and their families in the endemic villages of Sitapur, India.

In this study, we conducted semi-structured interviews with 22 participants, comprising both persons affected by the disease and their family members, using a 1:1 ratio. The findings revealed that stigma was pervasive, with stereotypes and negative beliefs about Hansen’s disease persisting in society. Participants, both patients and family members, reported experiencing various forms of stigma, which contributed to mental health issues such as anxiety and depression. A significant gap in knowledge about the disease existed not only among patients and families but also within the broader community and even healthcare providers. Social challenges such as reduced marriage opportunities were reported, although segregation was limited. Coping strategies like non-disclosure of the disease and self-distancing were commonly employed. However, family support emerged as a strong protective factor against the negative effects of stigma.

The study underscores the need for greater education and awareness to reduce stigma and improve mental health outcomes. Future research should focus on larger populations and assess the effectiveness of specific interventions aimed at combating stigma and promoting public knowledge about Hansen’s disease. More studies exploring the effects of stigma on persons affected by Hansen’s disease and their families should be conducted across other endemic geographical areas to improve the transferability of these results.

## Introduction

Mental health problems, mainly depressive symptoms and anxiety disorders are commonly reported among persons affected by Hansens’s disease [1]. Hansen’s disease is an old but treatable, chronic, infectious disease (caused by the bacteria *Mycobacterium leprae*) with a slow progression that gradually affects peripheral nerves and may lead to deformity [2,3]. Hansen’s disease is defined by the World Health Organisation (WHO) as a Neglected Tropical Disease (NTD) as it keeps emerging in few public health systems today: India is still among the most endemic countries attributing annually 60% of new global cases [3]. Hansen’s disease is closely linked to poverty, with stigma and disability pushing already disadvantaged individuals deeper into hardship [4]. Although the economy in India is rapidly growing nationally, poverty remains widespread [5]. A multi-centre cross-sectional study in four endemic states of India (Chhattisgarh, Maharashtra, West Bengal and Tamil Nadu) reported a prevalence of depression among persons with Hansen’s disease 33.1%, which is very high as compared to the 4.4 to 4.7% in the general population of India [6]. Likewise, they found a prevalence of anxiety of 19% compared to 3.0% in the general population [6].

Along with physical malformations, it has been hypothesized that most psychological problems result from the association with Hansen’s disease-specific stigma and discriminatory behaviour [1]. Bonkass et al. [7] conducted a systematic review of specific interventions addressing psychosocial challenges in persons affected by Hansen’s disease, offering insights into measures that could be adapted for implementation in India. Research indicates that anxiety toward Hansen’s disease is deeply rooted in Indian society, manifesting in an unjust fear of touching affected persons or anything they have come into contact with, such as food they have prepared [8]. In a scientific review, van Brakel et al. [9] outlined common misconceptions and fears associated with Hansen’s disease. According to the available findings, this fear is further reinforced by the misconception that Hansen’s disease is incurable [9,10]. The authors further explain that some persons believe that Hansen’s disease is either ‘bad karma’ resulting from bad behaviour in a previous life or a punishment from higher powers for a sin [8]. Consequently, as explained by the authors, persons affected by Hansen’s disease experience problems in their marriages or difficulties getting married and specific challenges in their jobs or obtaining employment [9]. As a result, their community interaction is affected, and even social relationships change [1,9].

Law systems have a detrimental role in protecting norms, defining socially acceptable behaviour and defending victims of human rights, highlighted by Carcianiga and Herselman in [11] an anthropological investigation of the history of Hansen’s disease. As explained by the authors, the legislation targeting persons with Hansen’s disease was meant to protect society from the risks of the disease rather than addressing problems and protecting persons suffering from the disease who need specialized care and guidance with social integration [11]. This discriminatory trend, instead of a protective attitude, is dominant in India [12].

Other factors impeding eradication in India are care-seeking behaviour, lack of awareness and treatment adherence [13]. Sermrittirong and van Brakel [14] describe in a review of the available research on Hansen’s disease that cultural beliefs and fear of discrimination are likely causes for persons to hide their symptoms as long as possible, even at the expense of the possibility of curing within 6–12 months relative to the type and severity of Hansen’s disease. This delayed-care-seeking behaviour hampers eradication efforts and increases the risk of negative physical and mental health outcomes for the individual, resulting in a vicious circle of illness, disability and discrimination [13,15]. Therefore, stigma is one of the causes of delayed-care-seeking behaviour and the interruption of treatment [16]. In a mixed-method study in an endemic state of Western India, it was illustrated that delayed care-seeking could result from a lack of awareness of the early symptoms of Hansen’s disease, therefore not seeing it as ‘threatening enough’ to visit a doctor and leading to recurrent late case identifications [13].

The family context, ignored in most studies and interventions into Hansen’s disease, has the potential to influence individual health outcomes [1]. In a systematic review of the effects of Hansen’s disease on mental health in persons affected by the disease and their families, Somar et al. [17] highlighted that persons affected by the disease are primarily affected by their diagnosis, yet it can also change the lives of family members. The authors show how the fear of discrimination in children can lead to psychological consequences, as the occurrence of low self-esteem and depressive symptoms, were reported in three studies within the review [17]. In addition, the previously mentioned review from van Brakel et al. [9] argues how family dynamics can influence personal decision-making, e.g., decisions about disclosure or care-seeking behaviour. Hence, family dynamics are relevant to understanding individual health outcomes. The authors also describe how the family can enforce stigma and exclusion toward persons affected by Hansen’s disease when the family decides, for example, not to provide support [9]. Thus, the available literature highlights how social exclusion or protection by social ties (families, community etc.) shapes stigma formation.

The current study explores the shared and distinct psychosocial challenges experienced bypersons affected by Hansen’s disease and their families using qualitative interviews. This study aims to answer the research question: ‘ How does stigma in society, including influence on public policies, affect mental health of persons affected by Hansen’s disease and their families in Sitapur, India?’ In exploring this question, this study also considered the role of care-seeking behaviour, treatment adherence and marriage dynamics/opportunities.. Depression and anxiety were taken as a proxy for mental health as they are the most reported among persons affected by Hansen’s disease in quantitative studies [1,5]. The four definitions of stigma used for this study were derived from the systematic review of stigma and mental health by Fox et al. [18]: experienced, internalised, anticipated and perceived stigma.

This study aspires to contribute to the knowledge needed to reduce inequalities among persons affected by Hansen’s disease by studying remote villages in an endemic district of India. Bonkass et al. [7] conducted a systematic review of specific interventions addressing psychosocial challenges in persons affected by Hansen’s disease, and highlight the importance of including the local context for tailoring intervention for specific populations of persons with Hansen’s disease. Further, as Hansen’s disease is a chronic disease, more attention is needed to facilitate the re-integration of persons affected by the disease in society. The findings may contribute to a better understanding of stigma and its role in the development of mental health issues, particularly depression and anxiety. The results will be of utility to program implementers to understand how to use their resources optimally. This study will use the term Hansen’s disease to refer to the disease caused by the bacteria Mycobacterium Leprae as the oldest name ‘Leprosy’ has received negative and stigmatising connotations [19,20]. Although this stigmatising term ‘leprosy’ is still widely used in India, we have deliberately decided to avoid it in order to promote stigma-free language in discourse surrounding Hansens disease.

## Methodology

### Ethics statement

Research was conducted in accordance with the Declaration of Helsinki. The GLRA India intervention study included the embedded studies and received local ethical clearance granted by the Institutional Ethics Committee (IEC) of Lepra Society, India (07/LEPRA IEC/2022).

The local research team created a list of potential participants through door-to-door visits after receiving ethical approval. Participants were informed about the study and asked to confirm their willingness to participate. Consent was obtained both orally and in writing using a format from the Lepra Society, tailored to the GLRA India study. If participants couldn’t provide written consent (due to deformities), they could use an ink stamp. The research team took care to explain the study thoroughly, ensuring participants understood the research purpose and their rights.

### Consent to participate

Written and oral informed consent was obtained from all participants before interviews . Participants were briefed on the study’s purpose, procedures, and their right to withdraw at any time without consequence. All consent procedures followed ethical guidelines and the Declaration of Helsinki. Participation was voluntary, and participants could refuse to answer any question or end participation at any time. By signing the consent form, participants agreed to have their interviews recorded and data used for research, with audio and transcriptions anonymized. A master list with unique codes linked participants to their data, which, along with all other data, was securely stored in password-protected files. Translators also provided verbal consent to ensure confidentiality.

### Study design

This study is part of the first phase of GLRA India’s larger intervention project, “Challenging stigma through engaging persons affected by Hansen’s disease in innovative methods of storytelling.” The goal is to empower those affected by Hansen’s disease in Sitapur, India, by reducing internalized stigma through storytelling. While the broader project involves a pilot cohort study with a mixed-method design, includes pre- and post-intervention measurements, this embedded study focuses exclusively on the pre-intervention phase. Using a cross-sectional design and qualitative methodology, it stands as an independent investigation conducted by GLRA India.

### Study site

The study was conducted in Sitapur, Uttar Pradesh (UP), India, with interviews held at the Panchayat Bhavan community center in Tappa Khajuria village. Participants were recruited from surrounding villages after obtaining official ethical clearance. The GLRA India research team selected this district due to their established, well-functioning team and the desire to begin their pilot intervention in a familiar location. Another key reason was the high prevalence of Hansen’s disease in the area.

### Study population, sampling frame and recruitment strategy

Participants were drawn from various villages in the district, based on district-level data of registered Hansen’s disease cases, without purposive selection. Using systematic randomization, 42 persons were selected and interviewed for the GLRA India intervention study. Inclusion criteria were: a Hansen’s disease diagnosis between 2020–2021, registration for MTD treatment, age over 18, and residence in Sitapur for at least one year. Disability grade was not an exclusion factor. Exclusion criteria included disabilities from other causes, illness, or unwillingness to provide informed consent.

The sampling frame for persons with Hansen’s disease was pre-selected by GLRA India for their intervention study, but only a subset was included in this study. Family members, however, were specifically recruited for this study through convenience sampling. The local research team invited family members accompanying patients to their appointments for interviews, and two additional family members were recruited by phone. Inclusion criteria for family members were living in the same district and being a first- to fifth-degree relative. In total, 11 family members participated, and only those related to the selected participants with Hansen’s disease were included, resulting in 11 participants from the GLRA India cohort.

### Questionnaire design

The interview guide was based on an online platform for qualitative research on Hansen’s disease, designed to support coherent research and questionnaire use. [20]. It adapted the “How to assess health-related stigma and mental well-being” interview guide and incorporated structured questionnaires, including the patient health questionnaire (PHQ-9) and the 5-question stigma indicator–affected persons (5-QSI- AP). The PHQ-9 is a commonly used and effective tool to measure depression and anxiety and was validated and approved for effectiveness in the Indian context [21]. The scale consists of two components: a cognitive and somatic component as these two groups of symptoms appear in clinical settings [21]. The 5-QSI- AP scale measures enacted, anticipated and internalised stigma and is recommended by the WHO in their monitoring and evaluation guide [22], however has not been validated in the Indian context. See S1 and S2 Texts for the interview guides for those affected by Hansen’s disease and family members respectively. Importantly, for the family members, the PHQ-9 questionnaire was included without adjustments. However, changes were made to the 5-QSI-AP scale to fit the context.

The interview guide contained five sections with different topics: (1) knowledge, attitudes and practices, (2) disclosure, (3) overall mental health, (4) stigma and (5) need assessments. The third and fourth sections included the two scales. The disclosure section and a question on family dynamics were added specifically for this study. Since participants could be illiterate, the scale questions were asked orally. For closed questions, no probing was done for “never” answers, but further questions were asked for other responses to gather more detailed information. [23]. All other questions in the guide were open-ended. Importantly, responses to the scales were not analysed quantitatively; rather they served as interview prompts to elicit qualitative data.

### Data collection

The semi-structured interviews were conducted between June 28th and July 1^st^ 2022. Recruitment began two weeks before the interview date and ceased on the day of the interviews. Before the interviews, participants gave consent and provided demographic data, including age, gender, occupation, education, income, household role, and, for those affected by Hansen’s disease, disease grade (WHO grade 0, 1, or 2). The two researcher from GLRA India, familiar with the study’s focus, conducted the interviews and asked follow-up questions. External researchers took field notes during the interviews. While some participants spoke Hindi, many used a local dialect, which posed challenges for the team. Translation support was provided by the local research team when necessary. Interviews were transcribed into Hindi and then translated into English for analysis.

### Data analysis

A thematic analysis, following Braun and Clarke’s method [24], was used to analyse the data. The Health Stigma and Discrimination (HSD) framework guided this study, acknowledging the role of the individual to the community and policy factors in stigma, as well as the possibility of both persons affected by a disease and their close contacts to experience stigma [25].

The interviews were coded based on HSD framework themes, with flexibility for iterative adjustments, hence a combined deductive/inductive approach [25,26]. Interviews were coded based on HSD framework themes, with flexibility for iterative adjustments. The coding scheme was refined through repeated readings of the interviews and field notes. Data analysis was conducted using ATLAS.ti version 22.0, with the same researcher overseeing all steps. See S3 Text for the final coding scheme.

## Results

The HSD framework guided the analysis, and thus the headings of the framework correspond to subheadings in the following section [25]. Among the subheadings, the themes that emerged while analysing the data are presented. All identified themes and sub-themes were organised in a code tree, see S3 Text for an overview. In the following sections of the results, we introduce the themes according to the different components of the framework, referred to as “layers”.

### Drivers, facilitators and ‘stigma marking’

**Fig 1:**
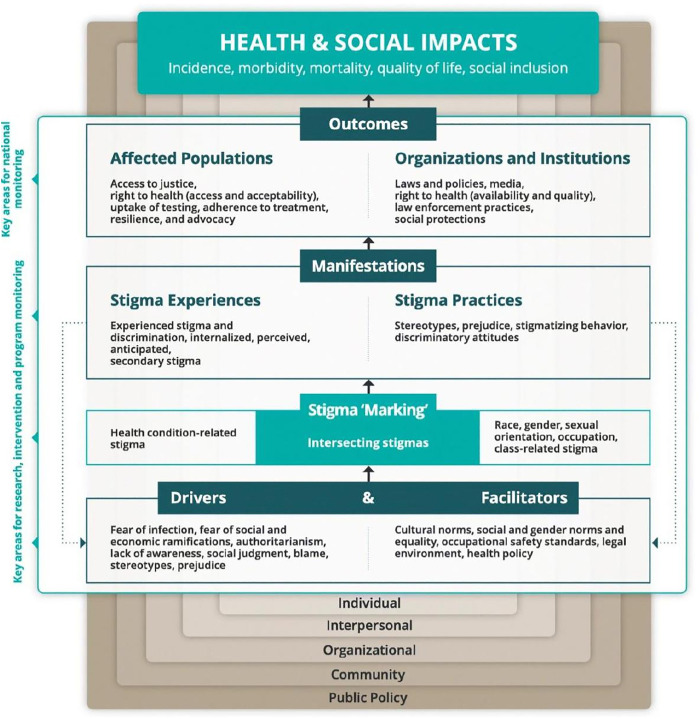
Health Stigma and discrimination framework [25].

To explain the first layer of the framework, drivers and facilitators together influence ‘stigma marking’ or the formation of stigma, belonging to the second layer. Drivers are always negative, while facilitators can be positive or negative. Drivers stimulate stigma, such as stereotypes or prejudice, while facilitators can facilitate or impede stigma, such as cultural norms or the legal environment (Fig 1).

Four themes were identified as drivers of stigma: *Insufficient or inaccurate knowledge*; *the presence of discriminatory behaviour and attitudes; fear of infection; and fear of social and economic ramifications*.

Under the first, *Insufficient or inaccurate knowledge*, three subthemes were distinguished: *name and characteristics disease unknown; nature infection unknown; inaccurate or wrong explanation diagnosis doctor.* Most participants (persons with Hansen’s disease and family members) had no prior knowledge about the disease before their diagnosis. In addition, they learned about the disease only upon contact with the doctor. Therefore, participants in this study did not find the right treatment in time, the following quote by one family member serves as a representation of commonly shared delayed-care-seeking behaviour due to a lack of knowledge of the disease:

‘*Earlier mummy used to get numb at this place if she pricked or pinched something, then she did not know, so we thought that something must have happened, then took medicine from the doctor in the village, then white spots started appearing, then the old people in the village told: ‘This is a symptom of Hansen’s disease’. Then we went to Sitapur to take medicine. In Sitapur, we were told that there is a hospital in Block Parsendi, and we got treatment from a doctor here.’*

#### Family member – Son of person affected with Hansen’s disease.

All participants (persons with Hansen’s disease and their family members) received treatment, and almost all believed the disease was curable, despite one person for whom the treatment did not seem to work.

Due to limited prior knowledge, the participants fully depended on the information provided by their doctor. However, doctors informed the persons affected by Hansen’s disease minimally about the condition and the consequences during treatment. Usually, only information about the treatment or care of physical symptoms, such as wounds, was given. Next to the seemingly limited counselling from the doctor, misinformation about the nature of the infection was common. Some participants described being given recommendations for distancing and advice on the separate use of utensils or other materials. The following quote from a person affected serves as an example of the misinformation given by doctors in the region:

*‘We came to know for the first time when the doctor told not to touch anyone and do not give food and drink to anyone.’*

#### A Person affected with Hansen’s disease.

The second theme, *the presence of discriminatory behaviour and attitudes* appeared central in the conducted interviews. In response to the question ‘What are the local beliefs in your community about this condition?’, different forms of prejudice, social judgement and blame were named. Many persons affected by Hansen’s disease and their family members reported the villagers believe the disease is contagious. In addition, they shared that community members think contact with persons affected by the disease should be avoided, which is indicative of poor knowledge in society as well. Thus, there seemed to be insufficient knowledge among persons affected by Hansen’s disease, family members, doctors and villagers, all driving (layer one; Fig 1) stigma in society (layer two; Fig 1).

The themes *fear of social and economic ramifications and fear of infection* appeared as drivers of stigma experiences and practices. All participants (persons affected and their family members) reported villagers were afraid to contract the disease, and at the same time, did not want to infect anyone themselves, as represented by the following quote from a person affected by Hansen’s disease:

*‘Interviewer - What do the people of the village think about this disease?*

*Patients - everyone runs away because of illness*

*Interviewer - What do they think, why don’t they touch you?*

*Patient- They thinks they will also get this disease, that’s why they do not touch’*

#### A person affected with Hansen’s disease.

At the same time, all the participants reported to fear the reaction of members of society. One family member appeared especially careful at the beginning of the interview due to this fear, as depicted by the following discourse between the interviewer and a family member:

‘*Interviewer- Can we record?*

*Family member - this will not be heard anywhere in society.*

*Interviewer - No, not at all, you trust me*

*Family member- This disease has to be hidden in society.’*

#### Family member – Son of person affected with Hansen’s disease.

Three themes were identified as facilitators of stigma marking in society: *decision to disclose the disease; trust in provided treatment; and familial support*. After being aware of the need for care, most participants showed straightforward care-seeking behaviour by initially seeking care from the general practitioner in the BCM hospital within the district. Once they received the diagnosis of Hansen’s disease, they had two options: either receiving it at BMC hospital but paying for it or going to the Precendi centre: the closest hospital to the district with free medicine from the government. Most persons choose to register for medication at the Precendi Centre. Many persons with Hansen’s disease talked about receiving free medications, which likely contributed to good treatment adherence among them. In addition, *trust in the provided treatment* was a common belief among persons affected by Hansen’s disease and their family members. The only reasons for interruption of treatment were practical, such as the lockdown during the COVID-19 pandemic. Participants reported the nearest facility with Hansen’s disease treatment was far away, which induced challenges, among which the fear that people in the village or outside understand they went for Hansen’s disease treatment. Many participants did not *disclose the disease* to villagers, which often led to the perceived good behaviour of villagers. Yet, in some cases, participants were forced to disclose the diagnosis due to the physical manifestations of the disease.

In most cases, the occurrence of the disease was told to all the family members within the same household, except for two parents hiding the disease from their children in fear of induced stigma. Most persons affected by Hansens’s disease said that their family members did not discriminate against them and received *support from their family members*, being it to a different degree: taking medicine, giving food and water, taking over work, etc.

### Stigma practices and experiences

After ‘stigma marking’ and application of stigma onto people, stigma manifests itself in stigma experiences or practices, which is the third layer of the framework. The lived stigma experiences include the four stigma categories (enacted, anticipated, internalised and perceived), while societal beliefs, actions and attitudes form stigma practices (Fig 1). Different themes were identified as stigma experiences: *experiences of physical distancing by others***;** *anticipation and fear of social consequences and feelings of embarrassment*. Under stigma practices, one major theme was identified: *discrimination against touch*. All stigma practices and experiences were related to health condition-related stigma (layer two) and no clear intersecting stigma was identified.

*Discrimination against* touch resulted in various stigma practices. First and foremost, it became apparent that there is a stereotype around Hansen’s disease in society as multiple participants shared the villagers think Hansen’s disease is a “touchable disease” and hold negative views on persons with “leper”. Consequently, villagers hold the prejudice that it is better to stay away from persons with Hansen’s disease and follow different discriminatory behaviours, such as refusing to sit next to persons affected. The following quote of a person with the disease and the interviewer provides an example of the stigmatising practices in society:

‘*Those people think that better to stay away. They have a very dirty feeling towards this disease. When we go to fetch water, they stand away from us, and then we feel very bad.’*

#### A person affected with Hansen’s disease.

This stigma experience (experienced stigma) by one of the participants directly shows how stigma practices are experienced by persons affected by the disease. Persons with Hansen’s disease and their family members shared similar stigma experiences (perceived stigma; stigma experience independent from individual opinions) related to the physical distancing by others in society. In addition*, feelings of embarrassment* (internalized stigma) for the disease were frequently reported among both persons affected by Hansen’s disease and their family members. In addition, the cultural norms and beliefs around the disease and its characteristics inflict fear on persons affected by Hansen’s disease and their family members. Participants repeatedly shared worries that persons would discover their diagnosis (or the diagnosis of their family member) as they expected and feared (anticipated stigma) the associated consequences of the stigma practices of the villagers. The following quote by a family member illustrates common thoughts among persons with the disease and their family members and represents the *anticipation and fear of social consequences*:

*‘Yes, we do all the work ourselves. And if someone comes to know about Hansen’s disease, the worry remains because people discriminate that if you stay near them, you may get Hansen’s disease.’*

#### Family member – Husband person affected with Hansen’s disease.

This quote immediately shows that stigma experiences are not limited to persons with the diagnosis but exacerbate further to the persons in their immediate surroundings (secondary stigma), the families. The interviews with family members revealed that family members can experience all types (experienced, perceived, internalized, anticipated) of stigma as a consequence of the diagnosis of their family member. In particular, cancelled weddings or fear of problems in marriage children were mentioned frequently by both persons affected by Hansen’s disease as their family members, showing again that the whole family can be affected by the diagnosis. The following quote is an answer to why the family decided not to disclose the disease to the neighbourhood, represents this common belief and fear:

*‘This is the reason people start discriminating, there is a problem in getting married, and people start saying don’t get married at their place.’*

#### Family member – Son of person with Hansen’s disease.

**Outcomes of stigma experiences and practices:** Stigma manifestations ultimately affect a wide range of outcomes for affected persons, being the person with the disease or the persons close to them via secondary stigma. For both the persons affected by Hansens’s disease as their family members, three themes were identified: *unable to work, psychological problems* and *coping strategies*. Many persons affected by Hansen’s disease experienced challenges with working, for a big part related to their physical symptoms. However, discriminatory behaviour also impeded the ease of finding and keeping work. Likewise, for family members the diagnosis of Hansen’s disease in the family induced disease-related stigma and challenges in conducting their usual work routine.

For the second, yet most important theme of this study, *psychological problems*, many subthemes were identified, such as *less interest in doing things* and *feelings sad or hopeless*, see S3 Text for an overview of all the sub-themes. Most of the sub-themes correspond with the PHQ-9 scale and all of the questions occurred in at least some of the participants. Most frequently, *feelings of sadness* and *less interest in doing things* or *less attention* were reported in persons with Hansen’s disease and their family members. *Loss of appetite* was also frequently reported and in some cases *trouble falling asleep or less energy/strength*, mainly in interviews with persons with Hansen’s disease but also with their family members. *Suicidal thoughts* were present only in persons with the disease, as two persons share:

*‘Yes, sometimes I used to think of dying, I should die so that can get rid of this disease.’*

#### Person with Hansen’s disease.

*‘The family members become dejected and sad. My nephew’s good marriage was fixed, but the village people informed (about the Hansen’s disease status) and the marriage was called off. I thought to lie down on the railway track and get killed when train come.’*

#### Person with Hansen’s disease.

Moreover, *tension in the mind* and *feelings of worry/anxiety* emerged as additional (to the PHQ-9 scale) themes in this study. Different factors were shared as causes of feelings of anxiety, such as worries for the future of the family, and worries about stigma and death), as shown by the following conversation between the interviewer and one of the participants:

‘*Patient- Sometimes when I am worried, I can’t sleep.*

*Interviewer- Is it often or sometimes*

*Patient- No, it doesn’t happen often, it happens sometimes, but we are worried that when we will not be able to work, what will we and our children eat?’*

#### Person with Hansen’s disease.

Some patients with Hansen’s disease depicted situations very typical for depressive symptoms:

*‘The mind remains sad. Sometimes get worried. Then do not feel hunger and thirsty and continue lying down whole day.’*

#### Person with Hansen’s disease.

Overall, persons with Hansen’s disease and their family members shared to feel less happy after the diagnosis.

Then, because of stigma experiences and practices persons with Hansen’s disease engaged in different strategies to deal with their diagnosis. The theme *coping strategies* were sub-divided into three themes: *(non-)disclosure of the disease*; *practice of self-distancing*; *and acceptance of diagnosis as part of destiny*. Among the persons with Hansen’s disease and their family members who reported fear of discrimination, non-disclosure of the disease was a common reaction, hence an outcome of stigma. The diagnosis was shared with family members (in most cases), yet the diagnosis was usually hidden from people in the village out of fear of the social consequences. In addition, persons with Hansen’s disease as well as their family members started to self-distancing themselves from other people in the village to avoid disclosure of the disease or (when disclosure already occurred) to limit the experiences of stigma. Many persons with Hansen’s disease and their family members mentioned not understanding why this disease was happening in the family and felt hopeless. In some cases, the diagnosis was accepted “as part of destiny” or “the will of god” yet psychological problems occurred also in these participants.

Moreover, specific to family dynamics, two themes emerged: *family situation affected* and *perceived distance between married couples.* First of all, the living situation of many families changed upon the diagnosis of Hansen’s disease. Despite the continued support, many families isolated or kept their distance from the person with Hansen’. In addition, the person with Hansen’s disease or the entire family lost connection with other family members (living somewhere else) and many persons with the disease reported thinking their family members feel sad, worried, disappointed, confused and dejected. Only one female person with Hansen’s disease reported their family becoming disheartened since her diagnosis:

*‘Interviewer: Have you ever felt bad about yourself or disappointed your family?*

*Patient- My family members sometimes become dejected because they are the head of the house, so they are bound to be dejected.*

*Interviewer - So you think you have disappointed the family*

*Patient- The family members have already become disheartened, if I get well then we will feel strong.’*

#### Person with Hansen’s disease.

The reaction of the family was not surprising to the woman, indicating that gender roles might be impacting the behaviour of family members to persons with the disease.

The diagnosis of Hansen’s disease showed to impact the marriage of persons as many persons with Hansen’s disease and their family members shared that there are more discussions at home and often physical distance, negatively impacting the relationship, as one husband explained:

*‘Interviewer- Has there been any change in married life between you two due to Hansen’s disease?*

*Family member- Due to Hansen’s disease, both of us keep a distance.*

*Interviewer - But how is the love between the two?*

*Family member- Love is not the same as before.*

*Interviewer: Has there been a change in the conversation?*

*Family member- Yes, there has been a lot of change in the conversation.’*

#### Family member – Husband of person with Hansen’s disease.

The husband was unaware of the fact they could return to old habits after completion of the treatment (even earlier). His expression changed positively when the interviewer told him.

A summary of the themes of the interviews mapped to the themes discussed above is presented below in Table 1.

**Table 1:** Themes emerged from the qualitative interviews in the current study, organised along four different layers derived from the HSD framework.

Layer 4		Outcomes of stigma	
	*Affected Populations*		*Organizations and institutions*
Themes	Individual-level (people with the disease and their family members): unable to work, psychological problems and coping strategies		
Themes	Family-level: family situation affected; perceived distance between married couples.		
**Layer 3**		**Stigma manifestations**	
	*Stigma experiences*		*Stigma practices*
Themes	Stigma experiences: experiences of physical distancing by others; anticipation and fear of social consequences and feelings of embarrassment.		Discrimination against touch
**Layer 2**		**Stigma ‘marking’**	
	*Health condition-related stigma*	*<intersecting stigmas>*	*Not conditioned related stigma*
	Hansen’s disease		(Poverty/gender norms?)*
**Layer 1**	**Drivers**	**&**	**Facilitators**
Themes	Insufficient or inaccurate knowledge, the presence of discriminatory behavior and attitudes; fear of infection; fear of social and economic ramifications.		Decision to disclose the diagnosis; trust in provided treatment, familial support.

*Factors hypothesized to be present among the included participants, yet not explored in depth in the qualitative interviews.

## Discussion

This study examined how stigma and its consequences impact the mental health of persons with Hansen’s disease and their family members, focusing on various types of stigma (internalized, experienced, perceived, anticipated, and secondary). It also explored the influence of care-seeking behavior, treatment adherence, and marriage dynamics. A combined deductive and inductive approach to thematic analysis was used, with the HSD framework providing structure and emerging themes drawn from the data. The findings, based on participants’ lived experiences, suggest that a lack of knowledge is the primary driver of stigma in society, resulting in both physical and mental consequences for persons with Hansen’s disease and their families.

Different stigma experiences emerged among persons with Hansen’s disease and their families in the remote villages of Sitapur. It seemed that most were aware of the idea of contagion and untouchability among the villagers, and most of the people with Hansen’s disease and their family members experienced fear of discrimination. In anticipation of the possible consequences of stigma, persons with Hansen’s disease and their close relatives feared social and economic consequences. Enacted or experienced stigma emerged mainly due to villagers (and one family) distancing from persons with Hansen’s disease and their family members, and this stigma emerged often as soon as those with Hansen’s disease cannot hide their disease due to disability. A feeling of embarrassment was a commonly reported finding, indicating that persons with Hansen’s disease and their families internalize the stigma. The findings of this study are in line with a part of the study findings by Kopparty (1995), who interviewed 500 patients and their families in two Indian districts and stated that patients experience shame or fear of isolation. In addition, even their families may experience or fear discrimination and reduced educational or job opportunities due to stigma, like the results of this study.

Yet, many misconceptions about Hansen’s disease exist in the cluster villages of Sitapur, and despite the fear of discrimination and belonging to a lower caste, family support was high, which conflicts with the results from [27]. Ideas of ‘untouchability’ resulting in fear of coming close to persons with Hansen’s disease prevail. However, unlike the findings of Kopparty [27] segregating persons with Hansen’s disease and their families from society did not occur, yet all participants did not (willingly) disclose the disease either. The presence of Hansen’s disease colonies in the region and the high manifestation of anticipated stigma in the analysed participants show that segregation still happens and inflicts fear on people. In a systematic review, Dalal [28] explains that illness is a family event in India, and familial support has been associated with coping with many chronic diseases, such as asthma or diabetes. In the current study, the supportive attitude of spouses seems to protect persons with Hansen’s disease from becoming isolated and could be beneficial to their mental health.

The findings of this study corroborate those of Heijnders [29], the author studied in a qualitative interview study anticipated and enacted stigma in the eastern region of Nepal and found that stigma is a dynamic process and happens in relationships with others. In addition, he explains that persons with Hansen’s disease can move from one stigma phase to another via multiple triggers, which can be the progression of symptoms or side effects. Coping strategies are central in his paper and he showed that persons with Hansen’s disease included in his study use these strategies to avoid moving from one stage to another, the most prominent being strategies of concealment induced by anticipated stigma. These strategies can include conjuring up ‘stories’ to retain social integrity. The author explains that once the symptoms progressed and concealment became more challenging, persons with Hansen’s disease developed new strategies, including withdrawing temporarily from situations where stigma could occur. This finding is similar to the non-disclosure and self-distancing as coping strategies anticipated among the participants in this study. However, other coping strategies, e.g., towards enacted stigma, were not explored in depth as the main focus was the link of stigma with mental health outcomes.

Moving to the mental health implications, the results show that stigma and the fear of stigma affect persons with Hansen’s disease and their families. A wide range of psychological problems emerged, hence indicating that anxiety and depression seem common among persons with Hansen’s disease and their family members included in this study, especially worry and fear for the future were frequently named. However, mental health appeared to be influenced also by other consequences of the diagnosis, especially physical symptoms in persons with the disease and the lower familial incomes due to work-related problems. Generally, many participants named poverty-related difficulties as their main concerns. Yet, regarding different sorts of symptoms, Grover et al. [30] conducted an extensive review of research on depression in India, with which he showed that while cognitive symptoms are also present among the depressive Indian population, somatic symptoms are the most common, indicating that the somatic problems (lack of sleep, less appetite and fatigue) found in this study could also be linked to depression. Also, the presence of suicidal thoughts highlights the impact on mental health in persons with Hansen’s disease, also reported in other studies [17,31]. More research will undoubtedly be needed to explore the specific reasons behind these mental events among Tappa Khajuria villagers as this was not questioned in depth in the current study. Furthermore, family members reported much fear and worry for the future and seemed to carry a burden as they needed to take care of all the familial responsibilities. This tension among family members highlights the need to include family members in research and intervention as the disease affects their lives too.

Delayed care-seeking behaviour is often reported as an obstacle to early diagnosis, increasing the treatment delay and risk of disability [13], also found among the participants in this study. Delayed care-seeking appeared as a determinant factor in a descriptive cross-sectional study by Samraj et al. [32] in UP, the same region. The authors describe that due to a lack of knowledge of Hansen’s disease and ignorance of the early symptoms, persons with Hansen’s disease often do not seek treatment until physical symptoms appear, often as a result of advice from people in their surroundings. Among the participants with Hansen’s disease in this study, physical symptoms and advice of others were crucial for seeking care, hence delayed care seeking due to lack of knowledge is likely. Future researchers interested in the relationship between care-seeking behaviour and individual health outcomes should increase the focus on the period between the first symptom appearance and the first appearance in a medical centre.

Another pertinent point discussed is the doctor’s responsibility to provide adequate information, which was often very limited among the villagers in the current study. In addition, the recommendations about distance are unjust as Hansen’s disease is not spread by touch, making such advice problematic, which was shown to impact marriage and family relationships, and likely contributes to the continuous formation of stigma in society. As Hansen’s disease could spread by droplets, it is necessary to avoid eating from the same food sources and distance initially. However, after starting treatment, persons with the disease typically lose infectivity within the first week of treatment [33], and continuous distancing for six months or longer is unnecessary. Samraj et al. [32] also mention the need to increase awareness among doctors and highlight their role in breaking the stigma as a doctor’s opinion or attitude towards a condition highly influences the perception of people towards it. Therefore, following the authors, it is essential to prioritize Hansen’s disease education in initial medical education and during a doctor’s professional training in later stages.

The persons with Hansen’s disease included in this study did not seem to have many problems adhering to the treatment. However, no questions were asked about side effects or other potential treatment issues. Most did seem to have much trust in the treatment and the possibility of a cure, which could positively affect mental health as it can reduce worry. However, this finding is in stark contrast with the main body of research on treatment adherence among persons with Hansen’s disease in India, where non-treatment adherence is usually a common finding among communities with low levels of Hansen’s disease-related knowledge [34]. Many persons with Hansen’s disease reported having problems with work and feelings of tiredness, which could be related to the side effects of the treatment [35], yet this was unexplored in this study.

The results show that Hansen’s disease-related stigma still affects marriage, increasing perceived distance between couples, and the marriage opportunities of children in families are at risk because of someone’s diagnosis, and the continued presence indicates that abolishment of a stigmatising policy is insufficient to reduce the stigma event happening in society. Looking at the HSD framework, stigmatising practices can expand further to organisations and institutions by, for example, inducing policy chang [25]. Hence, the various marriage acts were likely a consequence of stigma and reducing the discriminatory attitudes in marriage and marriage opportunities by taking away the facilitating policies, is one of the first steps. However, stigma removal will not succeed without addressing the lack of knowledge and misconceptions about the disease and all other factors contributing to ‘stigma marking’ in society [12]. Furthermore, Carcianiga and Herselman [11] state “It is obvious that prejudiced public perceptions of Hansen’s disease will not be completely eradicated until unfair legislation is replaced by positive norms and statutes protecting, rather than stigmatising, the victims of the disease”.

The findings correspond with the used HSD framework in which drivers, such as lack of knowledge or social judgement and facilitators, such as cultural norms, influence the’stigma marking’, and stigma manifestations impact various outcomes determining someone’s mental state. The HSD framework recognizes stereotypes and prejudice both as drivers of stigma and outcomes of stigma manifestations. However, the current findings indicate that more sections in the framework are not static. For example, anticipated stigma could stimulate a person to refrain from disclosing the disease. As a result, a person might not experience enacted stigma. However, disability might force disclosure, after which he might experience enacted stigma, possibly impacting individual (mental) health outcomes. Hence, this study showed that non-disclosure of the disease could be both a facilitator and an outcome of stigma experiences and practices.

### Strengths and limitations

A major strength of the current study was the involvement of persons with Hansen’s disease and their families. When stigma practices affect the whole family, including the family context in research is essential for the understanding of stigma formation in society and the development of appropriate interventions. The trusted local research team also played a key role, ensuring a 100 per cent response rate and preventing issues with mistrust. Their familiarity with the area, community, and culture facilitated logistics, contributing to the efficient completion of interviews within a week. Additionally, the GLRA India research team conducted the interviews, eliminating the need for external researchers. The positionality of the principal investigator also has the potential to influence the perspective with which the results are viewed.

The study has several limitations, notably the recruitment of family members and the integration of multiple research objectives into one interview guide. Family members were recruited based on their availability and willingness to participate, with a 100 per cent response rate, though this sampling method may introduce bias, as accompanying family members are likely to have supportive relationships. Additionally, some questions relevant to the study were asked differently or not posed to all participants due to overlapping research objectives. An unexpected language barrier also posed a limitation during data collection.

My perspective on the world differs from that of the district workers or participants in this study, as I come from an affluent country with a distinct culture and history. While the Indian researchers from GLRA India also have different backgrounds and cultures, they are more closely connected to the participants. By maintaining continuous awareness of these potential differences and critically assessing each step of the research process, I aimed to ensure that the lived experiences of persons with Hansen’s disease and their family members were represented as accurately as possible.

## Conclusion

This study supports findings from other research on stigma related to Hansen’s disease, coping mechanisms, and mental health issues among those affected. It also highlights that stigma burdens family members through experiences of stigma, anxiety, worry, and increased responsibilities, with marriage opportunities still impacted. Unlike other studies, this research found significant family support and minimal familial discrimination. A key issue identified was a lack of knowledge among persons affected by Hansen’s disease, their families, healthcare providers, and the community, which contributes to the ongoing stigma. These findings emphasize the need for context-specific research that includes the family, accounts for local dialects, and supports stigma reduction interventions, many of which have already been validated in India [36]. To break the cycle of stigma and poor health outcomes, policymakers should prioritize education to improve early detection and reduce discrimination. Further studies in other endemic areas are necessary to enhance the transferability of these results.

## Supporting information

S1 Text Interview guide persons with Hansen’s disease. (DOCX)

S2 Text Interview guide family members. (DOCX)

S3 Text Final code tree after thematic analysis. (DOCX)
