# Supplementary material for: Mental health and stigma in persons affected by Hansen’s disease and their families in rural Sitapur, India
Source: PLOS Ment Health. 2025 Nov 6;2(11):e0000475. doi: 10.1371/journal.pmen.0000475 (PMC12798344; doi:10.1371/journal.pmen.0000475)
Supplement: S1 Text — (DOCX) [file pmen.0000475.s001.docx]

**S1 Text: Interview guide persons with Hansen’s disease**

**Phase 1 Intervention Interview Guide**

**Participant Demographics-**

Name of the participant:

Age:

Sex:

Education:

Occupation:

Monthly Income:

Role in the household:

Grade of leprosy: (To be noted from medical records)

Date:

Study group:

**Introduction of the researcher**

“My primary goal is to see things the way you see them…it's more like a conversation with a focus on your experience, your opinions and what you think or feel about the topics covered in the questions I ask”

Would you like to participate in this interview?

Verbal Consent - Written consent -

Is recording OK?

**3. Background Information**

Overview: Can you tell me about yourself?

**Topic**

**Knowledge, Attitude and Practices about leprosy (only pre-intervention)**

- Can you tell me about your skin condition?
- How did you feel when you heard you were diagnosed with this condition? Can you explain why you felt this way?
- Before you were diagnosed, what were your thoughts on leprosy (skin condition)?
- Do you think it is curable? Why (not)?
- Who can get this disease (leprosy)?
- What are the local beliefs in your community about this condition?
- Where are you currently getting your treatment from?
  - How did you end up there?
  - Can you tell me about your help/treatment-seeking process from the time you got diagnosed to now?
  - Why did you prefer a local practitioner/practitioner further away?
  - How many practitioners did you consult before you got diagnosed?

**Disclosure (Stigma)**

- Have you told your family / friends / neighbours that you are affected by leprosy (skin condition)?
- Can you tell me about their reaction? How did you feel after you told them? Did you expect this reaction?

**Mental well-being**

- Do you have any difficulties with tasks because of your disease?
- Do you feel like the relationship with your family changed? Can you tell me about the change and how do you feel about it?

**Overall mental well-bei**n**g (probe further with WHY to receive detailed information) (To be measured before and after intervention)**

| **PATIENT HEALTH QUESTIONNAIRE-9**  **(PHQ-9)** | | | | |
| --- | --- | --- | --- | --- |
| **Over the last 2 weeks, how often have you been bothered by any of the following problems?** *(Use “*✔*” to indicate your answer)* | **Not at all** | **Several days** | **More than half the days** | **Nearly every day** |
| **1.** Little interest or pleasure in doing things | 0 | 1 | 2 | 3 |
| **2.** Feeling down, depressed, or hopeless | 0 | 1 | 2 | 3 |
| **3.** Trouble falling or staying asleep, or sleeping too much | 0 | 1 | 2 | 3 |
| **4.** Feeling tired or having little energy | 0 | 1 | 2 | 3 |
| **5.** Poor appetite or overeating | 0 | 1 | 2 | 3 |
| **6.** Feeling bad about yourself — or that you are a failure or have let yourself or your family down | 0 | 1 | 2 | 3 |
| **7.** Trouble concentrating on things, such as reading the newspaper or watching television | 0 | 1 | 2 | 3 |
| **8.** Moving or speaking so slowly that other people could have noticed? Or the opposite — being so fidgety or restless that you have been moving around a lot more than usual | 0 | 1 | 2 | 3 |
| **9.** Thoughts that you would be better off dead or of hurting yourself in some way | 0 | 1 | 2 | 3 |

**FOR OFFICE CODING**  *0*  **+** ______ **+** ______ **+** ______

**=Total Score:** ______

**If you checked off any problems, how difficult have these problems made it for you to do your work, take care of things at home, or get along with other people?**

| **Not difficult at all**  □ | **Somewhat difficult**  □ | **Very difficult**  □ | **Extremely difficult**  □ |
| --- | --- | --- | --- |

Developed by Drs. Robert L. Spitzer, Janet B.W. Williams, Kurt Kroenke and colleagues, with an educational grant from Pfizer Inc. No permission required to reproduce, translate, display or distribute.

**Stigma Indicator (probe further to receive detailed information) (pre and post intervention)**

**5-Questions Stigma Indicator – Affected Persons (5-QSI-AP)**

| **In the past year:** | **Never** | **Sometimes** | **Often / Usually** | **Don’t know** |
| --- | --- | --- | --- | --- |
| Have you experienced problems in finding or keeping work because you have (had) [condition]? | 0 | 1 | 2 | 0 |
| Have you been worried about others finding out you have (had) [condition]? | 0 | 1 | 2 | 0 |
| Have you felt ashamed because of your condition? | 0 | 1 | 2 | 0 |
| Have you had problems getting married / in your marriage because of [condition]? | 0 | 1 | 2 | 0 |
| Have people tried to avoid because you have (had) [condition]? | 0 | 1 | 2 | 0 |

**5-QSI-AP indicator score: 0-10**

**Assessing needs**

- What would help you in the future?
- What would have helped you in the past?

**End of Interview**

- Do you have any questions for me? For example, would you like to have more information on anything further?
- Is there anything else you would like to share with us?
