# Supplementary material for: Mental health and stigma in persons affected by Hansen’s disease and their families in rural Sitapur, India
Source: PLOS Ment Health. 2025 Nov 6;2(11):e0000475. doi: 10.1371/journal.pmen.0000475 (PMC12798344; doi:10.1371/journal.pmen.0000475)
Supplement: S3 Text — (DOCX) [file pmen.0000475.s003.docx]

**S3 Text: Final code tree after thematic analysis**

A Drivers and facilitators (Layer 1 HSD framework))

1. Drivers [d]:
   - Theme 1: insufficient or inaccurate knowledge [d]
     - Sub-themes [i]: name and characteristics disease unknown; nature infection unknown; inaccurate or wrong explanation diagnosis doctor
   - Theme 2: presence of discriminatory behavior and attitudes [d]
   - Theme 3: fear of infection [d]
   - Theme 4: fear of social and economic ramifications [d]
2. Facilitators [d]:
   - Theme 1: decision to disclose the diagnosis [i]
   - Theme 1: trust in provided treatment [i]
   - Theme 2: familial support [i]

B Stigma practices and experiences (Layer 3 HSD framework)

1. Stigma experiences [d]:
   - Theme 1:experiences of physical distancing by others [i]
   - Theme 2: anticipation and fear of social consequences [i]
   - Theme 3: feelings of embarrassment [i]
2. Stigma practices [d]:
   - Theme 1: discrimination against touch [i]

C Outcomes of stigma (Layer 4 HSD framework)

1. People affected: persons with the diagnosis and family members:
   - Themes 1: unable to work [d]
   - Theme 2: psychological problems [d]
     - Sub-themes psychological problems people affected with the diagnosis:
       - Less interest/pleasure in doing things [d]
       - Feeling sad or hopeless [d]
       - Trouble falling asleep [d]
       - Sleeping a lot [i]
       - Feeling tired, weak or dizzy [d]
       - Less or no appetite [d]
       - Feeling disappointed disease is problem in family [i] (not being able to work)
       - Attention diverted/not able to focus [d]
       - Walking/speaking more slowly [d] (to not be noticeable)
       - Suicidal thoughts [d]
       - Tension in the mind [i] (due to someone sick, no money, symptoms others)
       - Worries/anxiety (for future children, others coming to close) [i]
       - Less happy after diagnosis [i]
     - Sub-themes psychological problems family members
       - Less interest/pleasure in doing things [d]
       - Feeling sad or hopeless [d]
       - Trouble falling asleep [d]
       - Feeling tired, weak [d]
       - Less or no appetite [d]
       - Feeling disappointed disease is problem in family [i] (quarrel spouse, less income)
       - Attention diverted/not able to focus [d]
       - Walking/speaking more slowly [d] (to not be noticeable)
       - Tension in the mind [i] (due to someone sick, no money, symptoms others)
       - Worries/anxiety (for death, for future perspective, expenses) [i]
       - Less happy after diagnosis [i]
   - Theme 3: coping strategies [i]
     - Sub-themes coping strategies people affected with the diagnosis: [i]
       - Hiding disease (as long as possible)/(non)-disclosure disease [i]
       - Keeping distance on own/practicing self-distance [i]
       - Accept as part of destiny [i]
     - Sub-themes coping family members [i]
       - Keeping distance on own [i]
       - Accept as part of destiny [i]
2. Family dynamics:
   - Theme 1: family situation affected [i]
   - Theme 2: perceived distance between married couples [i]

[d] = deductive, based on the Health Stigma and Discrimination (HSD) framework, the patient health questionnaire (PHQ-9) and the 5-question stigma indicator–affected persons (5-QSI- AP)

[i] - inductive
